# How acceptable and usable is the Malawi maternal and neonatal COVID-19 surveillance platform? A mixed-methods implementation process evaluation

**DOI:** 10.1136/bmjopen-2025-111864

**Published:** 2026-11-11

**Authors:** Moses Kelly Kumwenda, Chikondi Chapuma, Deborah Phiri, Bertha Maseko, Chifundo Ndamala, Luis Gadama, Laura Munthali, Fannie Kachale, William Peno, Lumbani Makhaza, Andrew Likaka, Marc Henrion, James Chirombo, Rosemary Bilesi, Malangizo Mbewe, Martias Joshua, Clemens Masesa, Miriam Taegtmeyer, David Lissauer

**Affiliations:** 1 Institute for Resilient Health Systems, Liverpool School of Tropical Medicine, Liverpool, UK; 2 Helse Nord TB initiative, Kamuzu University of Health Sciences, Blantyre, Malawi; 3 University of Liverpool, Liverpool, UK; 4 Malawi-Liverpool-Wellcome Trust Clinical Research Programme, Blantyre, Southern Region, Malawi; 5 Data Management Support Unit, Malawi-Liverpool-Wellcome Trust Clinical Research Programme, Blantyre, Malawi; 6 Child Health Group, Malawi-Liverpool-Wellcome Trust Clinical Research Programme, Blantyre, Malawi; 7 Kamuzu University of Health Sciences, Blantyre, Southern Region, Malawi; 8 Ministry of Health, Lilongwe, Malawi; 9 Ministry of Health, Blantyre, Malawi; 10 Quality Management and Digital Health, Malawi Ministry of Health, Lilongwe, Malawi; 11 Medical Services, Malawi Blood Transfusion Service, Blantyre, Malawi; 12 Liverpool School of Tropical Medicine, Liverpool, UK; 13 Liverpool School of Tropical Medicine Department of Clinical Sciences, Liverpool, England, UK; 14 Malawi Ministry of Health, Lilongwe, Central Region, Malawi; 15 Department of International Public Health, Liverpool School of Tropical Medicine, Liverpool, UK

**Keywords:** COVID-19, Africa South of the Sahara, Pregnancy, Maternal medicine, Electronic Health Records

## Abstract

**Abstract:**

**Introduction:**

National surveillance systems improve the understanding of the magnitude of infectious diseases, their impacts on treatment outcomes, quality of care and thus enhance evidence-based decision-making and outbreak response. This study evaluated the process of implementing a surveillance system within the Malawian health system to understand how this health information intervention interacted with the context in building health system resilience.

**Methods:**

We conducted a mixed methods process evaluation investigating the establishment and implementation of a novel national maternal and neonatal COVID-19 surveillance system for Malawi from perspectives of healthcare workers. Quantitative usability data were collected by the research team from 33 health facilities in Malawi. Qualitative data was collected from four hospitals. A usability questionnaire was administered online to 40 healthcare providers who were directly involved in the implementation of the surveillance system to understand the appropriateness of the system. We used qualitative interviews involving 9 key informants and 14 healthcare providers to investigate the acceptability and sustainability of the system.

**Results:**

Most (34/40; 85%) study participants found the surveillance system easy to use, expressed confidence in using it (35/40; 87.5%) and perceived the system as being less cumbersome (37/40; 92.5%) and less complex (32/40; 80%) to use, demonstrating high usability and acceptability of the system. Qualitative data demonstrated the surveillance system was acceptable, useful in influencing facility decision-making processes through the availability of near real-time facility data and addressed data management challenges linked to paper-based systems. Despite these strengths, successful implementation was limited by systems technical difficulties, human resource problems and data collection challenges, highlighting the need for robust technical support and ongoing capacity building to ensure sustainability of the system.

**Conclusions:**

A national maternal and neonatal COVID-19 surveillance platform demonstrated high usability and acceptability among healthcare providers in Malawi. It also strengthened health information systems and demonstrated a clear capacity to improve facility decision-making processes. Full integration and long-term success of this surveillance platform require addressing technical difficulties, human resource constraints and data collection bottlenecks.

**STRENGTHS AND LIMITATIONS OF THIS STUDY**

- This study employed mixed-methods guided by implementation science framework to evaluate the implementation process outcomes of a novel national-level COVID-19 digital data platform in Malawi.
- Qualitative analysis was conducted in high and low performing health facilities limiting the interpretation of findings to facilities with intermediate performance.
- The study analysis fails to provide an impression of how the maternal and neonatal COVID-19 surveillance system would operate at the primary healthcare level within the Malawi health system.

## Introduction

 The COVID-19 pandemic imposed direct and indirect shocks and stressors on fragile African health systems inadequately prepared to respond and adapt to the pandemics, while maintaining essential services.^1^ Early reviews demonstrated that SARS-CoV-2 was associated with adverse pregnancy outcomes.^2 3^ In addition, increased maternal age, high body mass index, chronic hypertension and pre-existing diabetes were associated with severe COVID-19 during pregnancy.^4^ Furthermore, early research on indirect effects showed potential for COVID-19 to disrupt the delivery of comprehensive maternal and neonatal health services.^5 6^

A robust health surveillance system can effectively support the collection and prompt processing of information about direct and indirect effects of health emergencies. Selected African countries quickly developed COVID-19 surveillance but there were great variations in the level of implementation of the surveillance systems across countries.^7 8^ These variations could be attributed to inherent health information systems challenges in most African countries which are characterised by the lack of rigorous and robust data collection systems for general medical services due to challenges with internet bandwidth, limited computer resources and few numbers of trained personnel.^9^ Unlike developed countries, most African countries lack national electronic database systems specific to collecting indicators on emerging health emergencies but instead depend on routine household surveys to collect health indicators of interest in outbreaks.^10^ Using household surveys is unreliable for time-sensitive indicators during health emergencies. When prompt, accurate data collection, reporting and use are needed by decision makers, electronic data capture can fill this gap but requires adequate technical and infrastructural support, well trained personnel and sufficient financial resources to ensure sustainability of the system.^9^ It is critical for African governments to invest in national electronic database systems that are dependable, standardised and prompt data to support decision-making in health.^11^

Disease outbreaks create a window of opportunity for strengthening fragile health systems such as health information systems to improve patient care, enhance efficiency and optimise decision-making processes. Building responsive and resilient health information systems is important to access information and inform pragmatic decisions that would allow maintaining essential health services without compromising the quality and quantity during emergencies.^12^ A novel maternal and neonatal COVID-19 surveillance system (MATSurvey) was established to capture the direct and indirect effects of COVID-19 on maternal and neonatal outcomes within the Malawian health system. This study evaluated the process of implementing the MATSurvey to understand how the implementation of the system interacted with the context.

## Methodology

### The maternal and neonatal COVID-19 surveillance system

Malawi had its first three cases of COVID-19 on 2 April 2020 and in response,^13 14^ the Ministry of Health (MoH) and Malawi Liverpool Wellcome Research Programme (MLW) co-developed MATSurvey.^15 16^ The surveillance programme aimed to contribute towards national decision-making on maternal healthcare in a pandemic context and inform global efforts to understand and model the impact of COVID-19. Key stakeholders from the MoH made critical contributions in developing MATSurvey, ensuring that its design was consistent with the need. Involving stakeholders from MoH and public health officials from the districts through consultative meetings early helped identify key requirements and align the system with legal and cultural expectations. Through collaborative meetings, feedback sessions and transparent communication, stakeholders contributed to designing MATSurvey in a manner that balances functionality and accessibility.

The surveillance platform was rapidly rolled out by MoH and MLW in 33 health facilities across Malawi including all 26 government district hospitals (ie, 4 central hospitals and 2 district health offices) to document data on health system preparedness, quality of maternal and neonatal healthcare and severe maternal and neonatal outcomes.

The MATSurvey is an electronic platform that uses Open Data Software Kit (ODK) (OpenDataKit V.1.21.0) to collect data on COVID-19 maternal and neonatal demographic characteristics, clinical characteristics and outcomes. MoH’s Safe Motherhood coordinators coordinated implementation and collated data collected by health facilities and entered it into the computer tablet. Data committed to the database could be visualised near real-time by facility managers, healthcare workers and national decision-makers on a password-protected Zoho Analytics dashboard.

### Patient and Public Involvement

The development of the maternal health surveillance platform was directly informed by several unstructured consultations and engagement with representatives from health providers, policy makers and technical experts. Perspectives from these stakeholders informed the development and contextualisation of the maternal health surveillance platform, refinement of data collection approaches and interpretation of findings. Patients and members of the public were not directly involved since the platform was intended to be used by health providers. The study findings will be shared with participating stakeholders through accessible dissemination activities.

### MATSurvey implementation process

Jointly, the MoH and MLW implemented the MATSurvey through existing MoH structures and linking the platform to the maternal death and surveillance programmes. The system was introduced to health facilities from 27 July–5 August 2020. Participants included the Directors of Health and Social Services and their deputies from each district, labour ward staff (doctors and nurse–midwives) and data clerks from 30 districts across Malawi (see figure 1). MoH’s Safe Motherhood coordinators based at central and district hospitals took leadership of coordinating district-wide data collection during implementation. District teams captured routine paper-based data from ward handovers, ward-based handover books, theatre registers and labour ward registers using tablets. The oversight of the MATSurvey such as data management and technical support were provided by the MLW coordinating team. Three project staff, one per region (Northern, Central and Southern zones), worked with the MoH quality management directorate zonal officers to track data submissions progress and address system queries. MATSurvey project staff used a WhatsApp platform to provide a community of support for Safe Motherhood coordinators and enable shared learning.

**Figure 1:**
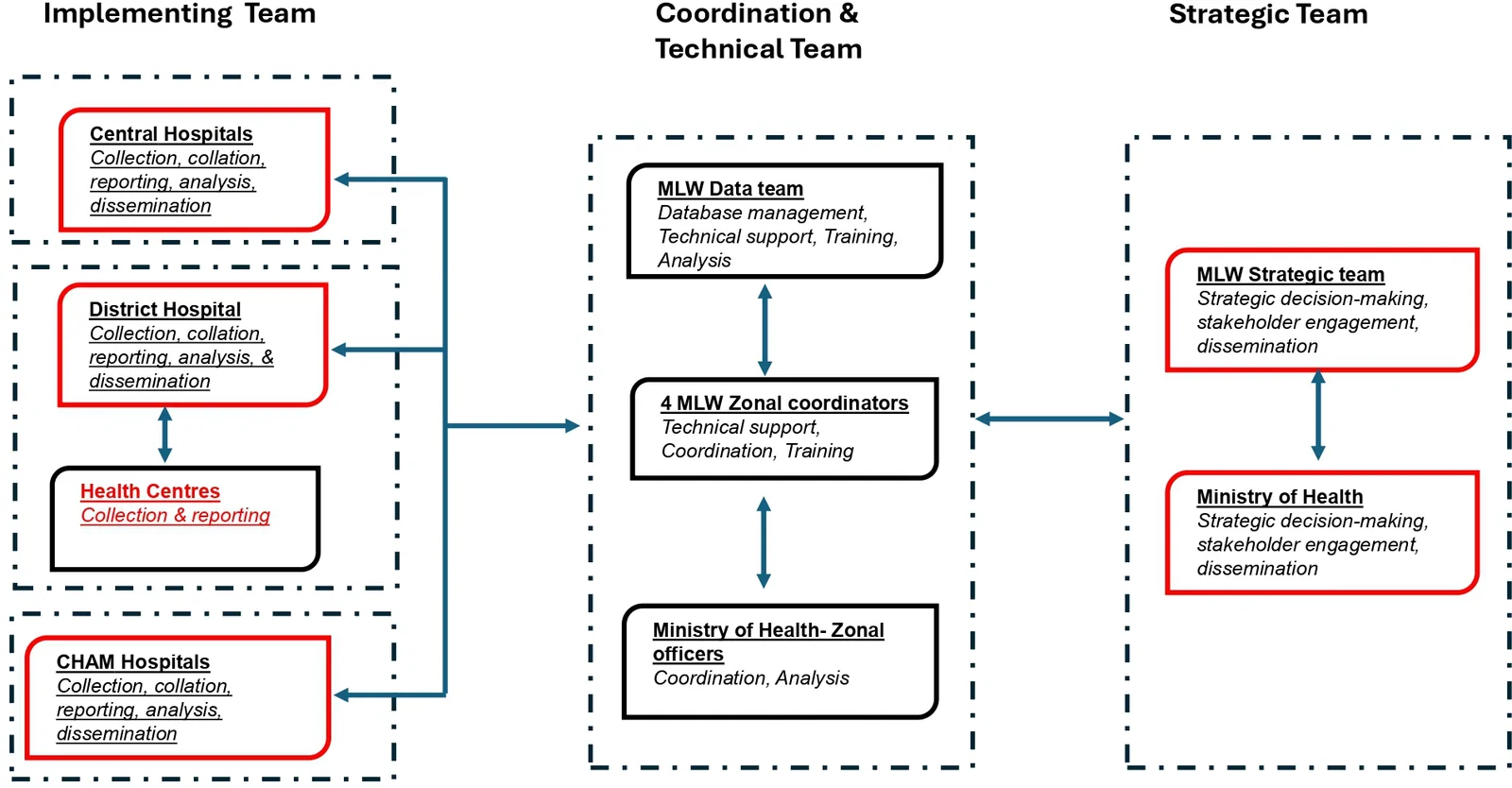
The MATSurvey implementation schema and data collection pathway. MATSurvey, maternal and neonatal COVID-19 surveillance system; MLW, Malawi Liverpool Wellcome Research Programme.

### Theoretical framework

The study applied Proctor’s taxonomy of implementation research outcomes to guide the evaluation of the MATSurvey system.^17^ The taxonomy identifies three domains of implementation research outcomes, namely (1) implementation, (2) service and (3) client outcomes (see figure 2).^17^ Our evaluation focused on the implementation processes of the MATSurvey with focus on acceptability, appropriateness, fidelity and sustainability of the intervention.

**Figure 2:**
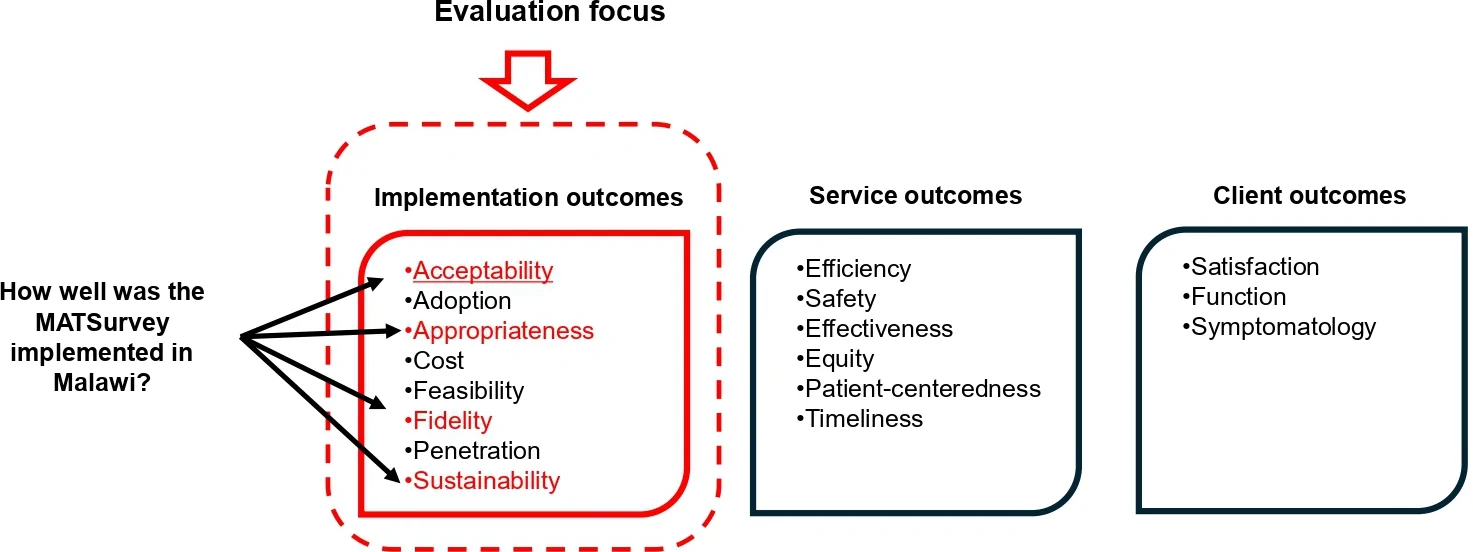
Proctor domains of implementation research outcomes. MATSurvey, maternal and neonatal COVID-19 surveillance system.

### Study design

A mixed-methods design (quantitative and qualitative) was employed to collect data on usability, acceptability, fidelity, appropriateness and sustainability of the surveillance system in Malawi and to understand the interaction between the intervention and the context. For the quantitative data, a 10-score usability questionnaire was self-administered to 40 end-users of MATSurvey from 30 health facilities. The questionnaire was developed by an MLW Data manager (DP) and sent via email to district teams involved in the implementation of MATSurvey. Additional quantitative data was extracted from the MATSurvey platform on the performance (fidelity) defined through completeness of data and timeliness of reporting. The Zoho dashboard’s access log was used to determine the access of information contained within the dashboard by users.

For the qualitative part, four hospitals participating in the MATSurvey were purposively selected based on their performance defined through timeliness of their reporting. We included two low-performing hospitals (ie, Mzimba and Bwaila district hospitals) and two high-performing hospitals (ie, Machinga district and Queen Elizabeth Central hospitals). 23 study participants were purposively selected for semistructured interviews (SSIs) with district teams (n=14) and key informants interviews (KIIs) (n=9). Guides for SSIs and KIIs were developed by the study teams and included questions on acceptability, appropriateness, fidelity and sustainability framed within Proctor’s taxonomy of implementation research outcomes (see online supplemental materials 1 and 2). Interview guides were also shaped by the study objectives. Data collection tools were pilot tested using peer researchers at MLW to optimise their ability to capture required information.

An experienced social scientist (MKK) and a public health scientist (CC) conducted the SIIs with individuals involved in the implementation of the MATSurvey between March and April 2021 (second wave). Each qualitative interview took between 54 min and 1 hour 30 min. Qualitative data collection ceased when point of saturation was reached—a point at which no new information was coming from data.^18^ Purposively selected members of the MATSurvey district team for SSIs included healthcare providers in the maternal and neonatal health departments, district data clerks, quality improvement officers and district health managers.

KIIs were also conducted with purposively selected individuals whose role involved the development of the surveillance platform of the implementation of the MATSurvey. These included actors embedded within the institutional hierarchies of MoH such as the Head of Quality Improvement Department, as well as academic experts from the Maternal Health Department at the Kamuzu University of Health Sciences. Additionally, members of the coordination and data management teams within MLW were engaged for their experiential insights into the operations and mechanics dimensions of the MATSurvey.

Quantitative usability data were explored through simple descriptive statistics, including percentages and proportions, and visualised using graphs and tables to convey patterns in user interaction. In parallel, qualitative data were captured using digital audio recorders to preserve the richness of participants’ narratives. Backup copies of the audio files were securely stored on Research Electronic Data Capture (REDCap) by the MLW data management team, while additional copies were shared with the transcription and translation team. The audio recordings were transcribed verbatim in Chichewa to retain the integrity of participants’ expressions before being translated into English for analysis.

Processed data were imported into NVivo V.12 (QSR, Melbourne, Australia) where they were organised according to document type to facilitate ordered exploration. Drawing on Proctor’s implementation outcomes framework, a hybrid coding approach—combining deductive code informed by this framework and literature with inductive categories appearing from data—was applied. This qualitative data coding process led by MKK yielded an analysis that was predominantly descriptive in nature but attentive to the multiple layers of meaning embedded within participants’ narratives. A 32 checklist of the Consolidated Criteria for Reporting Qualitative Research (COREQ) guided the reporting of these findings to ensure transparency and analytical rigour.^19^

Assessment of governance and legal compliance was undertaken by staff from MLW’s Clinical Research Support Unit, whose work reflects the institutional logics and regulatory frameworks shaping health research practice in Malawi.

## Findings

We begin the presentation of our findings with demographic characteristics of study participants before providing an overview of descriptive statistics that illuminates patterns in the usability of the MATSurvey. This is followed by an exploration of the socio-technical and institutional dynamics that shaped implementation, foregrounding the implementational enablers and barriers encountered within everyday practices and organisational landscapes for health systems actors.

Table 1 provides the demographic characteristics of participants included in the study. More female (n=29) than male (n=11) health providers completed usability questionnaire with the majority in the 31–40 years age group (n=24). Only female (n=14) health providers took part in SSIs with district teams and like the usability questionnaire, most participants were in the 31–40 age group (n=7). District teams had only female participants because the MATSurvey system was coordinated by Safe Motherhood coordinators, the majority of whom are female. Four male and five female participants were included as key informants from the coordination team.

**Table 1:** Demographic characteristics of usability study participants

Data collection approach	Sex		Age group				Total
	Male	Female	20–31	31–-40	41–50	51–60	
Usability questionnaire with district teams	11	29	12	24	4	0	40
SSIs with district teams	0	14	2	7	2	3	14
KIIs with Coordination team	4	5	3	4	1	1	9
Total	**15**	**48**	**17**	**35**	**7**	**4**	**63**

CHAM, Christian Association of Malawi; KIIs, key informants interviews; SSIs, semistructured interviews.

### Usability/appropriateness of MATSurvey system

Of the 40 district implementation team members directly involved in implementing the MATSurvey platform, the majority reported positive usability experiences. Most found the platform easy to use (34) and expressed confidence in their ability to use it (35). Key functionalities of the MATSurvey platform were perceived as well integrated (35); and 31 felt that other healthcare providers could learn to use the system quickly. A large number of usability participants (35) reported that they would like to frequently use the MATSurvey platform.

Moreover, the system was perceived as user-friendly: 37 disagreed that it was cumbersome, 32 disagreed it was unnecessarily complex and 28 disagreed that it had too many inconsistencies. Notably, however, 30 acknowledged that they needed to learn a lot about the platform and 24 needed some aid during use. Table 2 summarises responses to 10 usability questions.

**Table 2:** Summary of the responses to usability score questionnaire

System usability scale item	Strongly disagree n (%)	Disagree n (%)	Neutral n (%)	Agree n (%)	Strongly agree n (%)	Positive response n (%)
1. I would like to use the platform frequently	5 (12.5)	0 (0.0)	0 (0.0)	17 (42.5)	18 (45.0)	35 (87.5)
2. I found the platform unnecessarily complex	10 (25.0)	22 (55.0)	0 (0.0)	6 (15.0)	2 (5.0)	32 (80.0)
3. I thought the platform was easy to use	0 (0.0)	4 (10.0)	2 (5.0)	27 (67.5)	7 (17.5)	34 (85.0)
4. I would need assistance to use the platform in practice	3 (7.5)	10 (25.0)	3 (7.5)	24 (60.0)	0 (0.0)	13 (32.5)
5. The functions in the platform were well integrated	0 (0.0)	1 (2.5)	4 (10.0)	29 (72.5)	6 (15.0)	35 (87.5)
6. There was too much inconsistency in the platform	2 (5.0)	26 (65.0)	11 (27.5)	1 (2.5)	0 (0.0)	28 (70.0)
7. Most healthcare providers would learn to use the platform quickly	1 (2.5)	2 (5.0)	6 (15.0)	21 (52.5)	10 (25.0)	31 (77.5)
8. I found the platform cumbersome or awkward to use	10 (25.0)	27 (67.5)	0 (0.0)	3 (7.5)	0 (0.0)	37 (92.5)
9. I felt very confident using the platform	2 (5.0)	2 (5.0)	1 (2.5)	26 (65.0)	9 (22.5)	35 (87.5)
10. I needed to learn a lot before I could get started	0 (0.0)	9 (22.5)	1 (2.5)	23 (57.5)	7 (17.5)	10 (25.0)

In contrast to self-reported usability, an analysis of on actual MATSurvey utilisation portrayed low first engagement with the platform. Early data from the Zoho dashboard showed that by July 2021, only 100 of the 255 registered users (44.4%) had activated their accounts, leaving 125 accounts (55.6%) inactive. However, usage improved with time and by November 2021, 147 of 230 user accounts (63.9%) had been activated and 72.8% of these activated users were actively using the platform.

### Enablers and barriers to implementation of MATSurvey

District and coordinating teams identified key factors affecting implementation of the MATSurvey, which we have categorised as (1) facilitators and (2) barriers to successful implementation.

### Implementation enablers

Two distinct facilitators to the implementation of the MATSurvey platform appear: the platform simplicity and its capacity to address prevailing data management challenges.

### Simplicity enhanced acceptability of MATSurvey

KIIs and SSIs with platform users demonstrated high acceptability of MATSurvey. The system was perceived to simplify the collection of key maternal and neonatal indicators, including near-miss data previously excluded from the paper-based Maternal Death Surveillance and Response (MDSR) system. Users described the MATSurvey as uncomplicated and user-friendly, facilitated by its simplified digitised data collection approach via electronic tablets. MATSurvey permitted district health facilities to gather essential process data and generate descriptive statistics, supporting decision-makers in monitoring facility-level service delivery and implementing timely quality improvement measures. Overwhelming interest in the surveillance platform was also expressed across all levels of decision-making within the MoH and implementing partners in Malawi:

I think it is very much acceptable because it helps us to capture all the cases, … it may also help us know how we are doing as a facility in terms of maternal death issues… (Female, Senior Nursing Officer, Bwaila, MoH, Lilongwe)

The Quality Management Focal persons in the districts are very keen [in the surveillance system] … also district medical officers from all the districts*.* (Male, Quality Management Department, MoH)

Accessing district-level descriptive statistics from facility data was perceived as straightforward through the MATSurvey dashboards. Participants described that the MATSurvey allowed seamless, remote access to data for authorised users worldwide. They also highlighted that most data parameters collected by the system were already familiar to district teams, as they corresponded with indicators previously collected through the MDSR system.

…MATSurvey platform gives Safe Motherhood Coordinators the ownership of data compared with the system that they have been using before because nothing has changed in the data that they are collecting on daily basis. And, as a district, MATSurvey data when you create the data in the platform, you are giving feedback on the data that you have collected through PDF for dashboard. So, it is easy. (Female, Zonal Coordinator, MLW, Blantyre)

District teams prior experience with similar electronic data collection systems eased the smooth adoption of this innovation and supported its high-fidelity implementation.

### MATSurvey as a solution to existing data management concerns

Process evaluation participants perceived MATSurvey as a solution to persistent health systems data management challenges. Four key themes emerged and included (1) opportunity for transitioning to digital data management systems; (2) enhanced data security and quality; (3) improved fidelity through prompt feedback and (4) strengthened sustainability via system integration and capacity building through training.

#### Migration from paper-based MDSR system

MATSurvey was described as a timely, forward-looking and transformative=innovation for Malawi, that was responsive to global dynamics in information technology (IT). Its introduction facilitated Malawi’s shift from paper-based to an integrated electronic surveillance and reporting system.

…somehow, the system improved data documentation. If I can remember well, we had problems with paperwork. Most of the observations were not recorded. So, when we audited data, we usually found that there was a lot of missing data. (Female, District Nursing Officer, MoH, Machinga)

The demand for MATSurvey data was amplified among decision-makers from the MoH, particularly following a security concern that the District Health Information Software 2 (DHSII) encountered during the period of implementation.

… of late, we have been having several data requests, and I do not know if you heard about this, but the MoH lost data from to 2020 March up to September. So, we as MLW we provided some of this data … So at least, we are that that gap because they are missing data after September. We provided data for July to September… (Female, Data Manager, MLW, Blantyre)

#### Guaranteed data security and quality

Study participants perceived the MATSurvey platform’s robust electronic security measures as significant over the paper-based MDSR system was highly susceptible to tampering, loss and damage of data. The MATSurvey platform was also credited with enhancing user confidence in completeness and quality of collected data.

*…* The goodness is the safe keeping of the data that I enter, unlike if I store it in paper on report forms that are taken to HMIS [Health Management Information System] where they enter the information into DHISII… (Female, Data Clerk, Bwaila, MOH, Lilongwe)

#### Increased fidelity through prompt feedback

Study participants reported that MATSurvey permitted quick feedback of data and near real-time visualisation of facility-level outcome data. When data uploads or quality occurred, the data systems manager, through the zonal coordinators, promptly issued queries for district teams to resolve. This prompt management of data quality was perceived to enhance fidelity of implementation and ensured data completeness and accuracy. In contrast, such data appraisal and quality improvement processes were considered difficult, labour-intensive and time-consuming under the paper-based MDSR system.

…MLW zonal supervisors receive notifications from their respective zones after data is uploaded into the server … the data manager from the MLW will cross check if they are any duplicate forms or there are any queries in the system. It is also referred to us to respond to those queries but specifically the ones who communicate the queries …. (Male, Project Coordinator, MLW, Blantyre)

MATSurvey was perceived to increase data ownership among end-users by allowing district teams to proficiently track their data real-time and graphically visualise their facility’s performance through dashboards. The platform’s prompt graphical display of descriptive district-level analyses allowed prompt identification and rectification of data entry errors.

This platform can analyse the information presented on dashboard using these runs charts like the bar graph. It is really addressing some of the data issues which as a quality department we have been working for …. (Male, Quality Management Department, MoH) *-*

#### Assured sustainability through integration and training

Key informants highlighted MATSurvey’s strong potential for being sustainable, primarily because of its integration into routine MoH system from the outset. They underscored the critical role of engaging of strategic policymakers within MoH from conceptualization of the platform and the use of Safe Motherhood coordinators to lead implementation in the districts. However, they emphasised the importance of integrating the MATSurvey within the DHSII system to ensure long-term sustainability and prevent the proliferation of parallel data collection systems:

*…* I think … the DHIS system captures almost a lot of parameters within the facilities. I think this one [MATSurvey system] is more focused on maternal health parameters. There was that talk with the key people in the ministry on trying to see if we can link the data from this one to the DHSII system*.* (Female, Safe Motherhood Coordinator, Bwaila, MoH, Lilongwe)

Notably, efforts to integrate that MATSurvey within the DHSII had already commenced by the time of our data analysis. Participants further hinted that engaging the platform to leaders within the district health management teams and key strategic departments, such as Health Management Information Systems (HMIS), was valuable for sustaining this system. These stakeholders were viewed as holding decision-making authority and technical oversight necessary to ensure continued and effective implementation.

… I learnt you trained the management team, which is a plus. So, there are they… they know that this facility we are using this platform, this is a plus so sustainability will be there if you go. And then there is need to also train the members of the HMIS department. They can really assist in the sustainability of this tablet. (Female, Safe Motherhood Coordinator, Bwaila, MoH, Lilongwe)

### Implementation barriers

Implementation challenges of the MATSurvey were grouped into technical, health workforce and data collection domains, highlighting systemic and operational barriers.

### Technical challenges

Key technical challenges included poor network connectivity, rigid data submission protocols and limited flexibility in accessing or editing committed data.

#### Poor internet connectivity and system downtime

The MATSurvey platform depends on internet connectivity to transmit data to a server hosted at MLW, and poor network access posed significant obstacles to data transmission. Participants recounted that weak connectivity often hindered successful transmission of data. In such cases, the MLW data personnel followed up with respective district teams to resolve the problem. These connectivity challenges were particularly pronounced in certain districts within the Northern zone of Malawi:

For example, Likoma, I usually have problems … because of poor connectivity. (Female, Data Manager, MLW, Blantyre)

Another frequently reported technical challenge was ODK system downtime occurring due to malfunction within the system at MLW IT department. A system downtime meant that district teams could upload the data, but the system server would not receive and update the latest changes.

When you send the data, it brings a message to say it has not been successfully uploaded. This needs you to go to ODK to resend the data. … You go to save and then resend in the ODK. Just sending once it is not possible, it hangs somewhere called ODK. … (Female, Data Clerk, Bwaila, MOH, Lilongwe)

The MLW data management team acknowledged instances of ODK downtime but noted that these were limited to preliminary stages of MATSurvey implementation when the system had not fully been assessed and inherent technical issues not yet identified.

… on our end, the server downtimes. There was a time we used to have downtimes. In a week, I would say three or four times of downtimes. (Female, Data Manager, MLW, Blantyre)

#### Uploading incomplete data on the server

Safe Motherhood coordinators from both the low and high performing districts, along with the MLW coordinating team, reported challenges in uploading incomplete data. By design, the MATSurvey system restricted data upload onto the server if some fields in the case report form were incomplete. When this problem was found, the MLW data management team remotely accessed the affected district tablet to resolve the problem:

…we had put a restriction that the data cannot be pushed if it’s incomplete. This is something that the facilities have approached me. They give me a call that I’m unable to send the data. (Female, Data Manager, MLW, Blantyre)*-*

#### Inability to access committed raw data

Members of the district teams expressed frustration over their inability to access and correct the data after submission. Further frustration emerged because the system did not provide district teams with copies of raw data once uploaded:

…once the data has been sent, there isn't any way that they can make the corrections on the form …. (Female, Data Manager, MLW, Blantyre)

### Health workforce challenges

Two human resources-related challenges identified were training gaps and workforce instability.

#### Lack of training of members of district teams

District coordinating and data management teams concurred that limited training of healthcare providers was a key barrier to MATSurvey implementation. During data collection period, researchers observed that only a few individuals in four districts were familiar with the platform. Consequently, several health providers in health facility departments that supplied data to the MATSurvey were either untrained or insufficiently oriented, making data collection exceedingly difficult when trained staff were not available.

… other midwives also … are not competent to use the tablet. So, when the safe motherhood or the quality improvement focal person is off duty, then nobody enters the data. (Female, District Nursing and Midwifery Officer, Machinga, MoH, Machinga)

#### Staff shortage and turnover

High staff turnover and shortages posed a significant constraint to MATSurvey implementation. Some trained providers were frequently reassigned to other departments/hospitals through periodic rotation, transferred, retired or left for further studies. Successors often struggled to familiarise themselves with the electronic platform after a brief, on-the-job orientation. These on-the-job orientations were reported to be poorly managed, complicating transitions and hindering effective platform management.

Yes, but last, I think my colleague who do not have midwifery she has left for school, but she was also assisting me in other areas because she is working in HDU, so like near misses she would capture them, and we could assist each other. But currently I am just alone. (Female, Safe Motherhood Coordinator, MoH, Mzimba)

The coordinating and data management teams agreed that the inadequate training of members of district teams negatively affected quality of individuals’ interaction with the system, leading to reduced data quality and delayed submission:

… there were a lot of struggles to clean the data because there were issues which was down to training, people were not trained properly. There were times where the original person that was trained had left and there was no proper handover to the new person. (Female, Data Clerk, Bwaila, MOH, Lilongwe)

Involving additional staff within district facilities to support data collection processes proved very difficult as most staff were reluctant to participate without formal training or monetary incentives attached to training attendance:

…unfortunately, all my colleagues refused. They say that they have a lot of work … Female, Safe Motherhood Coordinator, MoH, Mzimba)

### Data collection challenges

District teams faced challenges collecting data from peripheral health facilities, including data on maternal death audits. Obtaining complete datasets required prompt submissions from multiple primary healthcare facilities linked to each district referral hospital. This process demanded frequent contacts with health centre staff who supplied the data, often involved frequent phone calls to all the health centres, sometimes funded personally by district team members. These problems usually delayed prompt submission of data:

…we are spending a lot of airtime calling people from health facilities reminding them … because you need … data from all the facilities. And when calling people, it is very hard. … we try our best to make sure that by Wednesday, all the facilities have submitted their data, … That is a big challenge. (Female, Safe Motherhood Coordinator, MoH, Lilongwe)

Several district facilities encountered challenges in reporting maternal death audits, especially because the numbers of actual maternal deaths reported in the district did not tally with the number of deaths audits conducted.

*…* the guideline for maternal death surveillance guideline it requires that the maternal death auditing should be done within7 days from occurrence. So, you find that some have passed 7 days, and they are still hanging unaudited*.* Female, Nurse Midwife, MoH, Lilongwe)

This process evaluation revealed discrepancies between what district teams accounted for maternal death audits and the data recorded in the MATSurvey. While the district teams reported conducting audits for all maternal deaths, the number of completed audits captured in the MATSurvey was notably lower. Participants from the two underperforming district hospitals insisted they consistently conducted audits but often relied on the old paper-based MDSR system, which explained the high number of unaudited maternal deaths reflected in the MATSurvey. Additionally, team members reported uncertainty about how and where to enter data on maternal deaths audits into the MATSurvey:

…they do not know where to upload the death audits in the tablet. I was once called and asked if I know how to upload death audit, I said no. Otherwise, it’s true that death audits here are done right away … (Female, Data Clerk, Bwaila, MOH, Lilongwe)

## Discussion

The WHO views surveillance as ‘the systematic ongoing collection, collation and analysis of data for public health purposes and the timely dissemination of public health information for assessment and public health response as necessary’.^20^ To our knowledge, this is the first paper to evaluate implementation process of a novel national-level and centralised COVID-19 digital data platform that systematically collected data on the pandemic’s impacts on maternal health outcomes in Malawi, provided descriptive analysis and disseminated processed information to decision-makers for use. Our quantitative findings demonstrate that the MATSurvey was appropriate for the Malawian context as healthcare providers it less complex, easy to use, aligned to existing systems and well-integrated withing pre-existing systems with less inconsistencies. As an upgrade to the paper-based MDSR systems, the MATSurvey addressed several data management issues associated with traditional reporting methods.

The MATSurvey system was highly acceptable and could be implemented with fidelity across Malawian hospitals. Healthcare providers found the electronic data capture, management and reporting platform easy to use, less cumbersome/awkward and confident to use it. Earlier studies on the usability of digital platforms during COVID-19 surveillance have focused on service provision and monitoring disease progression in patients,^21 22^ contact tracing^22 23^ and population-level outcome monitoring. This study offers a unique perspective by evaluating the entire implementation process especially within the maternal health context. Consistent with our findings, digital platforms used for infectious disease surveillance in other contexts have generally been well received by intended users.^24 25^

Adequate health workforce was critical for effective implementation of MATSurvey. While healthcare providers trained by the MLW’s coordinating team found the platform simple and easy to use, those introduced to the system through ad hoc on-job training often struggled to navigate this digital platform. Adebisi *et al* recognises the shortage of skilled human resources as a fundamental challenge undermining effective COVID-19 surveillance in Africa.^8^ Similarly, a COVID-19 surveillance system in South Africa encountered human resource problems, with some response teams often lacking epidemiology or surveillance specialists and revisions to the surveillance approaches were not consistently accompanied by added training and resources.^26^

This study observed a gradual but steady improvement in engagement with the digital system and the use of automated descriptive reports from the platform dashboards to guide decision-making processes at district health facilities. The availability of dashboards and automated reports strengthened and simplified interpretation of COVID-19 data. However, evidence of how district teams applied MATSurvey information to inform maternal and neonatal COVID-19 programming decisions is limited. This suggests potential underutilisation of the platform’s information for pragmatic action. Aborode *et al* similarly observed that the lack of knowledge among policy makers about the potential of the impact and influence of COVID-19 surveillance system on public health contributed to weak disease surveillance in Africa.^27^ By contrast, examples from other contexts such as Iran, show how near real-time surveillance system data supported important decisions during emergency situations such as resource allocation for hospitals, provision and distribution of equipment and funding, and setting up isolation centres.^28^

These findings also indicate that implementing MATSurvey system had a promise of being sustainable within the Malawian Health system. Strategic efforts to integrate the MATSurvey into the national DHSII system supported this sustainability. The platform’s co-development, co-design and nationwide scaled implementation were possible through active participation, support and contributions from stakeholders from multiple departments within MoH. As a result, within months of this evaluation, the MATSurvey was officially integrated within the Malawi DHSII system.^29^ Its subsequent use to assess the impacts of Tropical Cyclone Freddy on maternal health utilisation further proved the system’s added value to the Malawi Health infrastructure.^28^

MATSurvey implementation was hindered by the high staff turnover, limited opportunity for district teams to receive direct training from MLW coordinating team, and technical problems. Addressing these bottlenecks requires expanding the number of trained district team members and resolving recurrent technical problems. These challenges mirror problems findings from a 2013 study on improving district health systems using IT in five sub-Saharan African countries (Ghana, Mozambique, Rwanda, Tanzania and Zambia).^30^ Mutale *et al* reported barriers such as training and software, which required downstream interventions such as training all staff, recurrent face-to-face refresher training and using enhanced software.^30^ Supplementary challenges, such as poor internet networks, needed upstream interventions such as improving the quality of internet networks, engagement with internet network service providers and introducing electronic medical records. Similarly, poor internet connectivity was also reported as a key barrier to COVID-19 surveillance system implementation in rural Eastern Cape, South Africa.^26^

This study has several limitations. First, our qualitative analysis was conducted in only four purposively selected health facilities—two with high fidelity and two with low fidelity—limiting the interpretation of analysis to facilities with intermediate performance. Second, this analysis does not deliver an impression of how the MATSurvey would operate at the primary healthcare level within the Malawian context largely because of the design of the platform. Finally, we were unable to report on the performance of this system following the cutback of direct financial and technical support from the MLW coordination team and after integration within the national DHSII.

## Conclusion

Using Proctors implementation outcome framework, this paper reports that the MATSurvey digital platform for capturing data on the direct and indirect effects of COVID-19 on maternal and neonatal health outcomes was highly acceptable, appropriate and usable within the Malawian contexts. Fidelity during implementation varied from one district to the next and was partly influenced by high staff turnover and technical challenges which were rampant early during implementation. Lessons learnt in implementing the MATSurvey can inform policymakers and implementers where similar initiatives are being considered for implementation.

## Supplementary material

10.1136/bmjopen-2025-111864 online supplemental file 1

10.1136/bmjopen-2025-111864 online supplemental file 2

## Data Availability

Data are available upon reasonable request.
