# Supplementary material for: How acceptable and usable is the Malawi maternal and neonatal COVID-19 surveillance platform? A mixed-methods implementation process evaluation
Source: BMJ Open. 2026 Nov 11;16(11):e111864. doi: 10.1136/bmjopen-2025-111864 (PMC13756486; doi:10.1136/bmjopen-2025-111864)
Supplement: Supplementary file 1 [file bmjopen-2025-111864.reviewer_comments.pdf]

PEER REVIEW HISTORY

BMJ Open publishes all reviews undertaken for accepted manuscripts. Reviewers are asked to complete a checklist review form and are provided with free text boxes to elaborate on their assessment. These free text comments are reproduced below.

ARTICLE DETAILS

Title (Provisional)

How acceptable and usable is the Malawi Maternal and Neonatal COVID-19 surveillance platform? – A mixed-methods implementation process evaluation

Authors

Kumwenda, Moses Kelly; Chapuma, Chikondi; Phiri, Deborah; Maseko, Bertha; Ndamala, Chifundo; Gadama, Luis; Munthali, Laura; Kachale, Fannie; Peno, William; Makhaza, Lumbani; LIKAKA, ANDREW; Henrion, Marc; Chirombo, James; Bilesi, Rosemary; Mbewe, Malangizo; Joshua, Martias; Masesa, Clemens; Taegtmeyer, Miriam; Lissauer, David

---

VERSION 1 - REVIEW

**Reviewer**	**1**
**Name**	**Mdoe, Paschal**
**Affiliation**	**Haydom Lutheran Hospital, Obstetrics and Gynecology**
**Date**	**23-May-2026**
**COI**	**None**

I feel privileged to review a manuscript titled "*Building health systems resilience through strengthening Health Information Systems -A mixed-methods evaluation of implementing the Malawi Maternal and Neonatal COVID-19 surveillance platform*" The manuscript addresses important topic on health information system.

Generally, the manuscript is well written, however, the research question is "How was the implementation of the Malawi Maternal and Neonatal COVID-19 surveillance platform (MATSurvey) carried out, and what was its impact on strengthening health information systems and improving maternal and neonatal health outcomes in Malawi during the COVID-19 pandemic?"

The main findings were

1. The MATSurvey is highly acceptable, appropriate and usable in Malawi
2. 75% felt needed to learn a lot b4 using the system
3. Sytem usage improved over time
4. Qualittative highlighted enablers and Barriers

No where the impact is shown or supported by data, moreover, the conclusion is not based on the findings.

You may need to address this concern. Otherwise the paper is well writen

You may need to take into consideration the support and assess the post-support to assess usability of the system for sustainability

---

**Reviewer**	**2**
**Name**	**Tuladhar, Sabita**
**Affiliation**	**University Hospital of Munich**
**Date**	**27-May-2026**
**COI**	**None**

Thank you for the opportunity to review this paper. I have the following comments:

The title appears to be overstated. The paper is based primarily on perceptions, and there is no supporting data presented to justify the stated results or outcomes.

The manuscript is quite lengthy, which may affect readability and the ability to maintain the reader's attention.

Abbreviations should be expanded at their first occurrence.

There are several instances where words are missing or sentences are incomplete. The manuscript would benefit from thorough proofreading.

The Methods section is too brief and requires further detail. Tables 1 and 2, along with their findings, would be more appropriate in the Results section. Additionally, please clarify whether ethical approval was obtained.

For the quantitative component, the sample size is only 40; therefore, it would be more appropriate to present results in absolute numbers rather than percentages.

Page 15: Please clarify what is meant by "DHSII."

VERSION 1 - AUTHOR RESPONSE

Reviewer: 1
Dr. Paschal Mdoe, Haydom Lutheran Hospital, University of Stavanger Faculty of Social Science

Comments to the Author:

I feel privileged to review a manuscript titled "Building health systems resilience through strengthening Health Information Systems -A mixed-methods evaluation of implementing the Malawi Maternal and Neonatal COVID-19 surveillance platform" The manuscript addresses

important topic on health information system.

Generally, the manuscript is well written, however, the research question is “How was the implementation of the Malawi Maternal and Neonatal COVID-19 surveillance platform (MATSurvey) carried out, and what was its impact on strengthening health information systems and improving maternal and neonatal health outcomes in Malawi during the COVID 19 pandemic?"
The main findings were
1. The MATSurvey is highly acceptable, appropriate and usable in Malawi
2. 75% felt needed to learn a lot b4 using the system
3. Sytem usage improved over time
4. Qualittative highlighted enablers and Barriers
No where the impact is shown or supported by data, moreover, the conclusion is not based on the findings. You may need to address this concern. Otherwise the paper is well written You may need to take into consideration the support and assess the post-support to assess usability of the system for sustainability
Response: We sincerely thank the reviewer for the positive assessment of our manuscript and for this important observation. We agree that the original wording of the research question might have implied an evaluation of impact, but this was not what we wanted to achieve. Kindly note that the aim of this study was to evaluate the implementation process of the MATSurvey. In response, we have revised the manuscript to better align the study objectives, methods, results, and conclusions with the evidence generated. Specifically:

Our research question and study objectives focus on the implementation process of the MATSurvey platform and the evaluation of implementation outcomes, including acceptability, appropriateness, usability and factors influencing implementation

Please note that the word ‘impact’ in this manuscript is referring to the goal of MATSURVEY which was to capture information of the direct and indirect ‘impacts’ of COVID-19 on maternal outcomes. The Discussion and Conclusion emphasise that the MATSurvey platform demonstrated high acceptability, appropriateness, improving usability over time, and identified key facilitators and barriers to implementation, all of which are important implementation outcomes.
Regarding sustainability, we agree that assessing post-implementation usability would provide valuable evidence. However, this evaluation was conducted during the implementation period, and a formal post-support assessment was beyond the scope of the current study. We have acknowledged this as a limitation. We believe these revisions have substantially strengthened the internal consistency of the manuscript and ensured that the conclusions accurately reflect the evidence presented.

Reviewer: 2

Comments to the Author:
Thank you for the opportunity to review this paper. I have the following comments:

Comment: The title appears to be overstated. The paper is based primarily on perceptions,

and there is no supporting data presented to justify the stated results or outcomes.
Response: The title of the paper has been revised. It now reads: 'How acceptable is the Maternal and Neonatal COVID-19 surveillance platform? A mixed-methods study in Malawi'

Comment: The manuscript is quite lengthy, which may affect readability and the ability to maintain the reader's attention.
Response: Thank you for this comment. We acknowledge that the manuscript is relatively lengthy. The scope of the study, which includes multiple data sources and a comprehensive mixed-methods evaluation, necessitated detailed reporting to ensure transparency and methodological rigor. Nevertheless, we have revised the manuscript to improve readability by reducing redundancies, tightening the narrative, and enhancing the organization of key sections.

Comment: Abbreviations should be expanded at their first occurrence.
Response: Thank you for this helpful suggestion. We have carefully reviewed the manuscript and ensured that all abbreviations are now fully expanded at their first occurrence, followed by the abbreviated form. This has been applied consistently throughout the text to improve clarity for readers.

Comment: There are several instances where words are missing or sentences are incomplete. The manuscript would benefit from thorough proofreading.
Response: Thank you for this important observation. We have thoroughly proofread the manuscript and corrected instances of missing words and incomplete sentences. The revised version has been carefully checked to improve clarity, grammatical accuracy, and overall readability.

Comment: The Methods section is too brief and requires further detail. Tables 1 and 2, along with their findings, would be more appropriate in the Results section. Additionally, please clarify whether ethical approval was obtained.

Response: Thank you for this constructive feedback. We have revised the Methods section to provide additional detail, including clearer descriptions of the study design, data collection procedures, and analysis approach, to improve transparency and reproducibility. We have also merged Tables 1 and 2 into 1 (Table 1) and moved it along with their associated narrative, to the Results section, where they are more appropriately situated. In addition, we have clarified that ethical approval for the study was obtained, and the relevant details have been included in the last paragraph of Methods section.

Comment: For the quantitative component, the sample size is only 40; therefore, it would be more appropriate to present results in absolute numbers rather than percentages.
Response: Thank you for this helpful suggestion. We agree that given the relatively small sample size (n = 40), presenting results using absolute numbers improves interpretability and avoids overemphasis on percentages. We have revised the Results section accordingly, reporting findings primarily in frequencies while retaining percentages only where appropriate for clarity

Comment: Page 15: Please clarify what is meant by “DHSII.”
Response: Thank you for this comment. We have clarified the meaning of “DHSII” at its first occurrence in the manuscript to ensure that readers are fully informed.
