# Supplementary material for: How acceptable and usable is the Malawi maternal and neonatal COVID-19 surveillance platform? A mixed-methods implementation process evaluation
Source: BMJ Open. 2026 Nov 11;16(11):e111864. doi: 10.1136/bmjopen-2025-111864 (PMC13756486; doi:10.1136/bmjopen-2025-111864)
Supplement: Supplementary file 3 [file bmjopen-16-11-s002.doc]

**Supplemental material 1: Interview topic guide for District Teams**

**Introduction**

1.	Tell me about yourself, position- What do you do? [Getting to know interviewees background, What does a normal day look like? How long have you had this occupation?]

**Experience with the Maternal Surveillance System**

2.	What role did you play in the implementation of MATSurvey?

3.	How was the maternal covid-19 platform introduced to you?

4.	How does the Maternal and Neonatal Surveillance system currently work? Take me through different of how the system works. Probe: Where? Who collects data? How is data collected and from where? How does the facility use or interpret their data?

5.	How do you screen for eligible patients?

6.	What have been your experiences with the implementation of the maternal covid-19 surveillance platform in your district (Probe: acceptability of the platform, appropriateness of the platform)

7.	What are the main challenges that you face when using the platform? Probe: Data collection, Data use/interpretation. Probe: What have been the technical and implementation problems that you have experienced during the implementation of the maternal covid-19 surveillance platform?

**Impact of the platform**

8.	What changes has the introduction of the maternal covid-19 platform brought you to your health facility? (Probe: Benefits for the platform)

9.	How can the positive changes that the system has introduced be sustained?

**Future direction**

10.	How can we improve the implementation of this platform to ensure that important information is being captured at the right time?

11.	How can the implementation of the maternal covid-19 surveillance platform be introduced to the health centre levels?

12.	In what ways has the data captured by the platform informed clinical practice in your district?

13.	How can the maternal covid-19 surveillance platform be sustained?
